# Describing complex clinical scenarios at the bed-side: Is a systems science approach useful? Exploring a novel diagrammatic approach to facilitate clinical reasoning

**DOI:** 10.1186/s12909-016-0787-x

**Published:** 2016-10-10

**Authors:** Saroj Jayasinghe

**Affiliations:** Department of Clinical Medicine, Faculty of Medicine, University of Colombo, Kynsey Road, Colombo, 8 Sri Lanka

**Keywords:** Complex cases diagnosis systems science clinical reasoning map

## Abstract

Clinicians often encounter patients having complex clinical scenarios (CCS) where diverse and dynamic diagnostic and therapeutic issues interact. A limited range of bedside methods are available to describe such patients and most often it is a diagnostic summary, a problem list, or a list of differential diagnoses. These methods fail to portray the interconnected nature of CCCs. They prevent visualization of a system of networks or a web of causation operative in CCSs.

A more holistic conceptualization is required and the author argues for an approach based on systems science. The latter views the human body to consist of several closely linked organ systems, constantly interacting with each other and embedded in, and ‘open’ to the external environment. In order to capture the systems nature at bedside, a tool based on network diagrams, termed a Clinical Reasoning Map (CRM) is proposed which depicts diseases or conditions as nodes linked to each other by lines or arrows. The latter linkages follow simple rules: possible causes or associations as mere lines; probable cause using a single dotted arrow with directionality (from ‘cause’ to ‘effect’); definite causal pathways by directional arrows; and bi-directional arrows to indicate organs-systems influencing each other.

CRM’s utility was investigated in several groups of undergraduate medical students. The results varied: 289, 5th year and 4th year medical students showed that 245 (85.5 %) perceived CRM improve their understanding of the case. However, there was no clear advantage in the CRM over a list of diagnoses in recall of key information. A majority (83.9 %) were keen to learn the technique of drawing a CRM. Postgraduates too found the tool to be useful to understand the interconnected nature of real-life complex case scenarios and pathogenesis of their multifaceted condition to generate differential diagnosis and to select appropriate investigations. Effectiveness of CRM is supported by adult learning theories such as meaningful learning and experiential learning.

The author proposes that systems science and tools based in this approach such as CRM has utility in understanding and managing complex case scenarios. They differ significantly from other diagrammatic methods available in the medical literature.

## Background

Clinicians at bedside or doing office practice are required to tackle patients with a wide spectrum of clinical conditions, of which a significant proportion fall into an ill-defined category called complex clinical scenarios (CCS). These are scenarios with a collection of diverse and dynamic diagnostic and therapeutic issues which most often interact with each other. They tend to overlay other sets of psychosocial issues which further influence management and are often tackled by the other members of a multidisciplinary team.

CCSs are common in both acute and long term patient care. It includes multi morbidity: elderly with disorders that impinge on the function of several organ systems and those with an acute illness in the presence of an underlying chronic disorder. Similarly, young patients with diseases with multisystem involvement (e.g. HIV-AIDS with infections in multiple organ systems) would face the same issues. Coexistence of an acute illness in the presence of an underlying yet active chronic disorder is another situation: acute dengue with thrombocytopenia and fluid leakage in a patient on anticoagulants for a valve replacement [1]. These situations make clinical diagnoses, investigations and treatment difficult due to the dynamic interactions across several dysfunctional organs.

A limited range of methods are available to clinicians to describe patient related problems including CCSs, often in the form of a diagnostic summary or a problem list or a list of differential diagnoses. Other examples include extended summaries authored by multidisciplinary teams and case reports published in medical literature.

## Proposal

The author proposes that a systems science approach is required to tackle CCSs and describes a tool based on this approach (namely Clinical Reasoning Map) to understand and manage CCSs. The argument is unfolded in three steps: The limitations of existing tools used by clinicians for clinical reasoning; a system science approach to conceptualize clinical reasoning of complex cases; a novel bed-side tool based on systems science: Clinical Reasoning Map

### Tools used by clinicians for clinical reasoning and their limitations

A systematic method to document clinical problems that include diagnoses was popularized in the 1960s using a Problem Oriented Record Systems (PORS) [2]. This method allowed a continuous record or a list of active and resolved issues to be updated by a multidisciplinary team. It allows large volumes of data to be available to the clinician in the form of a summary, enabling detection of important issues. It is widely used in computer-based electronic medical records and useful to generate diagnosis specific registries [3]. More importantly, electronic clinical decision support systems often depend on these clinical problem lists [4–6]. PORS have also introduced the SOAP format (subjective, objective, assessment, and plan) which is useful to track the management longitudinally and to generate medical summaries [7–10].

The common feature to all the methods mentioned above is summarizing medical diagnostic information with the use of lists. The advent of electronically digitized records in health systems, often with computer interfaces those have drop-down menus may have consolidated the use of such lists to describe CCSs [11]. The usage of lists fails to portray the interconnected nature of the complex disease states. They are devoid of a mention of pathogenesis. As a result, the dynamic nature of several interacting disease states, the role of dysfunctional organ-systems, or a web of causation operative in CCSs is rarely portrayed. This may lead clinicians to consider individual problems or issues, in the expense of understanding the pathogenesis or interactions or inter-relationships. Therefore, a simple tool that would meet these needs is a dire necessity for busy clinicians in modern day practice.

### A system science approach to conceptualize clinical reasoning of complex clinical scenarios

An appropriate conceptual basis is required to develop a tool to tackle CCSs at the bedside. One recently proposed approach is systems science, which perceives events and conditions in the human body in terms of a system [12]. Briefly, this views the human body to consist of several closely linked organ systems (or subsystems) that are embedded in and ‘open’ to the external environment (e.g. through gas exchange during respiration, food intake and thermal interactions). These organ systems are constantly interacting with one another through diverse pathways such as chemical interactions (e.g. lactate produced in peripheral tissues being metabolized by the liver), direct diffusion of chemicals across tissues (e.g. development of a sympathetic effusion from cytokines released from a sub-diaphragmatic abscess), hormones (e.g. release of cortisol during sepsis leading to hypercatabolic states), immune pathways (e.g. release of antibodies from a infection leading to glomerulonephritis), and cytokines (e.g. release of tumour necrosis factor from an inflammation that results in a febrile response), to name a few. Normal or deranged function of one organ system influences many other organ-systems (and the reverse in the form of positive or negative feedback). A disease state is an emergent property of this system and is unique, though commonalities exist in many areas, and hence we see such patterns which we term or define as a ‘disease’.

### A novel bed-side tool based on systems science: Clinical Reasoning Map

Based on the concepts of systems science, the author has developed a simple tool to use at bedside [13]. It is a network diagram (i.e. a Clinical Reasoning Map or CRM) that supplements lists of differential diagnoses or problem lists and acts as an aid in clinical management. The nodes could be disease entities, disorders or clinical features, while arrows or lines indicate the inter-relationships across them (see next section on ‘Principles of drawing the CRM’).

The term Clinical Reasoning Map signifies that the tool is useful for clinical reasoning, which is defined as “the cognitive process that is necessary to evaluate and manage a patient’s medical problems” [14], These cognitive processes have a relatively slow analytical and deliberative process (i.e. the hypothetico-deductive approach) and a non-analytical rapid process that are unconscious and intuitive and involves pattern recognition [15], A map indicates multiple connections across areas of interest and the tool helps to trace the path of a patient’s condition as it evolves. It can be used as a deliberate pathway towards reaching a point or as a pattern of interconnections that are visualized almost instantaneously by the expert clinician.

CRM represents the interconnectedness of CCSs and is a visual representation of systems science at bedside. The lack of a strict hierarchy (as in concept maps) or a central point (used in Mind Maps) enables the clinician to show the evolution of the CCS over time. Nodes in the network are used as clinical entities (e.g. diseases or clinical features) and arrows to show the associations, pathogenesis or causality.

The next section describes the principles of drawing CRM, its utility in education, and a comparison with other diagrammatic methods used in medicine.

#### Principles of drawing the CRM

**Fig. 1:**
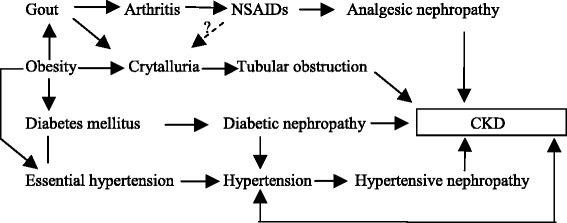
Clinical Reasoning Map of patient developing CKD. Possible associations (); probable cause (); more definite causal pathway (); bi-directional arrows to show disorders influencing each other (); uncertain links flagged by a question mark (see text for details)

The tool was initially proposed in 2009 to promote learning in medical students [16], Subsequently the term CRM was used to indicate its utility during the process of clinical reasoning and to reflect the web of causation of disease states [13]. The description below is an elaboration of this method. In a nutshell, it is a visual explanatory model of clinical scenarios using a systems science approach. The following example illustrates a 55-year-old male who had chronic kidney disease (CKD). His clinical features include obesity, gout, chronic analgesic use for gout, prostatism due to benign prostatic hyperplasia, poorly controlled type II diabetes mellitus and essential hypertension for 13 years. The underlying disease processes to explain CKD include diabetic nephropathy, obstructive uropathy, urate crystal nephropathy, analgesic nephropathy and hypertensive renal damage. This conventional list of problems or diagnoses or pathogenic mechanisms could be written down before developing the CRM (Fig. 1). However, with experience the clinician may produce a CRM *de novo*.

Certain conventions are used to draw the CRM. Connecting arrows or lines are used to show linkages between nodes (e.g. diseases).

- Possible associations: a line without arrow heads () such as the association of hypertension with diabetes.
- Probable cause: a single dotted arrow with directionality. () In this example, one may hypothesize that analgesics form a crystal-induced nephropathy to worsen CKD, as seen with ciprofloxacin [17],
- A more definite causal pathway such as diabetes leading to diabetic nephropathy: It is shown by an arrow with directionality. ().
- Bi-directional arrows to indicate organs-systems influencing each other. (). This is used to show the link between CKD and hypertension, which worsen each other.
- Any of the links that are uncertain is flagged with a question mark on top

This simple categorization of arrows makes the clinician explicitly state his or her assessment of the linkage. By convention, the pathways are ‘positive’ in valence and inhibitory pathways can be shown with a clear negative sign. (Not shown in figure).

#### Utility of CRM in undergraduate and postgraduate learners

The utility of CRM was investigated in several groups of undergraduate medical students previously [13]. Studies on 289, 5th year and 4th year medical students showed that 245 (85.5 %) perceived CRM to be useful to improve their understanding of the case, because it clearly presented the linkages and summarized information. However, there was no clear advantage in the CRM over a list of diagnoses in recalling the key information related to complex clinical scenarios. A majority of students (83.9 %) were keen to learn the technique of drawing CRM.

An evaluation of effectiveness of CRM as a tool in clinical reasoning was done on 11 postgraduate students, who had completed their internship, worked for more than one year as a medical officer, and passed an entry examination to the postgraduate training programme in medicine (i.e. MD). The entry examination consists of multiple choice questions, single best response, and objective structured clinical examination of clinical skills and could be considered as equivalent to the Membership of the Royal College of Physicians (UK) which now designed to assess clinical knowledge and skills of trainees wishing to enter higher specialist training. The participants were given a short presentation on the principles of clinical reasoning and on the utility of CRM (approximately 30 min). This was illustrated with two examples that showed a stepwise approach to the process. After a brief discussion, the subjects were made to individually draw a CRM on a single paper case, which was subsequently discussed in the group. This was followed by an individual task of drawing a CRM on a real patient currently in the ward (30 min). These were discussed in the wider group. Five days later, individuals were requested to select their own CCS and develop the diagrams and present to the group for discussion (1 h). Nine completed both sessions. On completion of the two meetings, perceptions of the participants were assessed using a self-addressed questionnaire. All found it useful or very useful to understand the interconnected nature of problems patients have, and pathogenesis of the illnesses and disorders. There was unanimity in CRM being useful or very useful in generating differential diagnosis and in selecting appropriate investigations.

#### CRM in relation to learning theories

The theoretical basis of CRM broadly fit constructivism, which views human knowledge as been constructed by individuals (or social communities) rather than ‘discovered’ or is passively absorbed [14]. The interpretation of CCCs using a systems science approach by the clinician is an example of this process where diverse entities are linked to synthesize or construct a holistic picture. The utility of CRM in understanding is explained through ‘meaningful learning’ and cognitive theory of multimedia learning [18, 19]. The ability of CRM to link a collection of clinical events adds meaning by relating new information to those already known to the learner (i.e. meaningful learning) and explains why the majority of undergraduates and postgraduates found it to help understand CCCs. Further studies are required to explore the utility of CRM in retaining such meaningful information in the long-term memory. CRM uses a diagrammatic method where words are linked by a set of conventions that add dynamism. This complement lists of differential diagnoses and reflects the principles of multimedia learning, i.e. words and pictures together which helps to produce logical mental constructs, promoting deeper learning than from words alone [19]. CRM is a tool that enables reflection and abstract conceptualization of CCCs. These are components of the experiential learning cycle described Kolb where experience are recognized and transformed through the cycle of ‘concrete experience, reflective observation, abstract conceptualization and active experimentation [20].

#### Comparison of CRM with other diagrammatic methods used in medicine

Algorithms are perhaps the commonest graphic method used in clinical medicine. They are defined as stepwise paths which aid diagnosis and treatment and take readers through predetermined dichotomous criteria and pathways, where decision nodes take the role of specific criteria [21, 22]. Algorithms are ubiquitous as posters which summarize guidelines and also available as textbooks [23]. However, they are linear, prescriptive and, useful to resolve a single diagnostic or a therapeutic issue. Concept maps are used as a graphic tool to organize and represent knowledge in a hierarchical manner and are effective in teaching/learning [24–27]. This contrasts with CCS which are best described in a non- hierarchical network with multiple interconnections. Ishikawa diagram is another tool that has been modified to assist clinical learning, but its utility is mainly to recall relevant aspects of the literature in relation to a case [28]. Mind Maps has found utility in depicting complex situations in medicine and other fields of education [29]. However, it requires a central concept with branching out of related concepts, limits its usefulness in describing clinical encounters which tend to evolve over time [30–32]. Causal diagram is another method used in population health and epidemiology to summarize information and specify causal relationships in the area of population health [33]. It differs from traditional epidemiology in that pathways that depict potential causes are the main focus, an approach established in other disciplines such as air pollution modelling [34, 35]. This could be described as an explicit attempt to take a systems approach to view health issues of populations. Recently this approach is being used in research programmes in urban health and wellbeing [36].

In summary, a wide range of diagrammatic methods are used in health sciences to deepen our understanding of events or assist in learning, yet they are rarely used at the bedside by clinicians and of limited utility at the bedside.

## Conclusions

The author argues that a diagrammatic method, namely Clinical Reasoning Map, based on a systems science approach is an useful tool to understand and manage CCSs. There are at least three reason ns in support of this stand. Firstly, the existing methods fail to capture the inherent interacting disease processes seen with CCSs, such as those seen with metabolic syndrome [37–39]. The growth of an elderly population and multimorbidity means CCSs would become increasingly common in hospital and clinic practice [40]. Secondly, there is preliminary evidence that learners perceive it as an useful tool to improve understanding of CCSs. This was outlined in the previous section in studies conducted on undergraduates and postgraduates. Thirdly, utility of CRM in understanding is explained through theories such as ‘meaningful learning’ and cognitive theory of multimedia learning. It is also a tool for reflective practice and abstract conceptualization. CRM resonates with other diagrammatic methods used in medical education, clinical practice and population health, yet has significant advances in its structure which improves utility to tackle CCSs.

## Abbreviations

CCS: Complex clinical scenarios
CKD: Chronic kidney disease
CRM: Clinical Reasoning Map
PORS: Problem Oriented Record Systems
SOAP: Subjective, objective, assessment, and plan
